# Postharvest Disease Management of ‘Akizuki’ Pear in China: Identification of Fungal Pathogens and Control Efficacy of Chlorine Dioxide

**DOI:** 10.3390/jof11100694

**Published:** 2025-09-25

**Authors:** Haichao Jiang, Lixin Zhang, Yang Zhang, Yudou Cheng, Cunkun Chen, Yongxia Wang, Junfeng Guan

**Affiliations:** 1 College of Life Science and Food Engineering, Hebei University of Engineering, Handan 056000, China; jhc20000819@163.com; 2 Institute of Biotechnology and Food Science, Hebei Academy of Agricultural and Forestry Sciences, Shijiazhuang 050051, China; feiliuzhao@163.com (L.Z.); taloyament@163.com (Y.Z.); chengyudouyn@163.com (Y.C.); 3 Key Laboratory of Postharvest Physiology and Storage of Agricultural Products, Ministry of Agriculture and Rural Affairs of P. R. China, Institute of Agricultural Products Preservation and Processing Technology (National Engineering Technology Research Center for Preservation of Agriculture Product), Tianjin Academy of Agricultural Sciences, Tianjin 300384, China; cck0318@126.com

**Keywords:** *Pyrus pyrifolia* ‘Akizuki’, postharvest diseases, storage, pathogen identification, chlorine dioxide

## Abstract

The ‘Akizuki’ pear has become increasingly popular in China in recent years. However, the ‘Akizuki’ pear often suffers from severe rot diseases during the postharvest storage period. Those during storage have not been thoroughly elucidated In this study, fungal pathogens causing postharvest decay of ‘Akizuki’ pear were identified through multi-gene phylogenetic analysis, followed by assessment of the antifungal efficacy of chlorine dioxide (ClO_2_) at varying concentrations. A total of 18 strains were isolated and identified as pathogens by Koch postulates. The isolated pathogens were taxonomically identified by combining morphological characterization of hyphae/spores with multi-gene phylogeny (ITS, *β-tub*, *tef1*). The results revealed that isolates A1-A11 were identified as *Alternaria alternata*, D1-D3 as *Diaporthe eres*, P1 as *Penicillium citrinum*, and P2-P4 as *Penicillium expansum*. The strain with the strongest pathogenicity in each genus was selected as the representative strain for subsequent control experiments. ClO_2_ significantly inhibited the development of the *D. eres*, *A. alternata*, and *P. expansum* by suppressing mycelial growth and disrupting cell membrane structure of pathogens, in which the EC50 values were 35.56 mg/L, 24.71 mg/L, and 41.98 mg/L, respectively, showing comparable antifungal activity to conventional fungicides. This has clarified the occurrence and control of postharvest decay diseases of ‘Akizuki’ pear fruit and provided more options for the practical applications in postharvest disease control of pear fruits.

## 1. Introduction

‘Akizuki’ pear (*Pyrus pyrifolia* Nakai) is a mid-to-late maturing sand pear variety, rich in various organic acids, soluble sugars, and multiple phenolic compounds essential for human health [1], and is highly favored by consumers for its uniform fruit shape, tender flesh texture, and sweet flavor [2]. China is the world’s largest producer of the ‘Akizuki’ pear, with an annual production of 1,500,000 tons, covering an area of about 80,000 hectares. In recent years, it has been widely planted and is currently predominantly distributed in areas such as Wei County and Zhao County in Hebei, Laiyang city in Shandong, and Xinyi city, Suining, and Tongshan city in Jiangsu.

However, the ‘Akizuki’ pear is susceptible to pathogen infections that lead to fruit rot. These problems seriously affect the quality and commercial value of the ‘Akizuki’ pear [3]. According to a 2023 report by the USDA, postharvest diseases of pears in China cause an annual yield loss of approximately 8%, with the economic losses exceeding USD 1.5 billion. Postharvest disease is a significant global challenge [4]. As a hallmark of postharvest disease, latent infection of fruit involves pathogen colonization at field development stages, while symptomatic decay manifests exclusively during postharvest storage under conducive conditions [5]. Fungal pathogens are causal agents of postharvest diseases in pears, such as mold diseases caused by *Penicillium* spp. [6], black spot disease by *Alternaria* spp. [7], soft rot by *Rhizopus* spp. [8], gray mold by *Botrytis cinerea* [9], rot disease by *Diaporthe* spp. [10], ring spot disease by *Botryosphaeria dothidea* [11], and rot disease by *Fusarium avenaceum* [12]. To date, there have been no reports on the identification of postharvest diseases in the ‘Akizuki’ pear. Conventional morphological identification only provides a preliminary classification of pathogens. With the rapid advancement of molecular biology techniques, molecular identification methods have come to be widely used for pathogen classification. Phylogenetic analysis using concatenated sequences offers a potential solution to addressing the classification of fungal species within genera [13].

Current control methods mainly rely on fungicides, such as triazole, imidazole, and benzimidazole [14]. However, excessive application of chemical agents results in environmental pollution, food safety concerns, and the development of drug resistance [15]. Therefore, there is an urgent need to find a green control method for postharvest disease control. In this situation, chlorine dioxide (ClO_2_) treatment presents a promising alternative. As a strong oxidizing agent, ClO_2_ has shown safe and effective characteristics in disease control, and has been granted approval by the Food and Drug Administration (FDA) in U.S. for postharvest application in 2004 [16]. Importantly, almost all residual ClO_2_ in edible tissues degrades into chloride ions, minimizing residue concerns. As a result, ClO_2_ is widely used in the control of the postharvest diseases of fruits. ClO_2_ can inhibit foodborne pathogens such as *Escherichia coli* [17] and *Listeria* [18] and significantly inhibit fruit rot [19] and the development of gray mold [20,21]. In addition, ClO_2_ can also alleviate enzymatic browning reactions in fruits [22]. As for the antimicrobial mechanism of ClO_2_, it shows that ClO_2_ treatment inhibits the growth of *P. digitatum* by disrupting cell membrane integrity [23], and enhances the chitinase and glucanase activities of fruits, leading to damage to the cell walls of pathogenic fungi [24]. Despite the widespread application of ClO_2_ in suppressing the postharvest diseases of fruits and vegetables to extend shelf life, ClO_2_ fumigation for controlling postharvest diseases in the ‘Akizuki’ pear remains unexplored.

To address the previously uncharacterized pathogenesis of the postharvest decay of the ‘Akizuki’ pear, this study systematically isolated and identified the pathogens causing rot in ‘Akizuki’ pears during the storage period, mainly isolating *Alternaria*, *Penicillium*, and *Diaporthe*. The sensitivity of the most virulent strains in each genus to ClO_2_ was determined, and the antifungal mechanism was investigated with the aim of providing a basis for in-depth research on the occurrence and control of postharvest diseases in the ‘Akizuki’ pear.

## 2. Materials and Methods

### 2.1. Fruit Collection

‘Akizuki’ pear fruits at maturity stage were harvested from 4 regions including Zhao County (114.896689 E, 37.775025 W), Wei County (115.2666089 E, 36.9820674 W), Ningjin County (114.9145405 E, 37.6180744 W), and Jinzhou City (115.0341055 E, 38.0328936 W) in Hebei Province, China. Then the fruits were transported to the laboratory, and stored at 0–1 °C. During cold storage, fruits that developed diseases were selected for the isolation, purification, and identification of pathogens.

### 2.2. Pathogen Isolation and Purification

A small piece of tissue was cut from the area at the junction between diseased and healthy parts, and then it was disinfected with 1% (*w*/*v*) NaClO for 1 min, followed by three rinses with sterile water. The tissues were plated on potato dextrose agar (PDA) medium and cultured in the dark at 28 °C for 8 days. Mycelia from the edges of the colonies were transferred to fresh PDA for purification. This process was repeated three to four times to obtain pure strains, which were stored in test tubes at 4 °C.

### 2.3. Koch’s Postulates

Healthy pear fruits were selected and disinfected with 75% ethanol. The fruits were divided into 18 groups, each containing six fruits. By using a sterile punch, a small hole (5 mm in diameter) was made on the equatorial surface of each fruit. After the wound dried, a 5 mm mycelial plug was obtained using a sterile punch and inoculated into the puncture site, whereas treatments without fungal isolates served as controls. All samples were then kept in a constant humidity incubator (Shanghai PHC Health Medical Devices Co., Ltd., Shanghai, China), maintained at 25 °C with 90% humidity. The diameter of the lesions was measured on days 0, 2, 4, 6, and 8, respectively.

### 2.4. Morphological Identification

The purified *Penicillium* and *Alternaria* were cultured on PDA medium and the purified *Diaporthe* on oat agar medium (OA) according to Bastide et al. [25]. All cultures were incubated in the dark at 28 °C for 8 days. Conidia and mycelia were mounted in water and examined under an optical microscope (OLYMPUS BX 51, Tokyo, Japan). The experiment was repeated 3 times.

### 2.5. Molecular Biological Identification

The mycelium was collected from the media of the isolated strains. The total DNA of 18 strains was extracted using a fungal genomic DNA rapid extraction kit (Sangon Biotech, Shanghai, China). Primers ITS1/ITS4 and Bt2a/Bt2b were used to amplify the sequences of strains P1, P2, P3, and P4 [15]; primers ITS1/ITS4 and Bt2a/Bt2b were used to amplify the sequences of strains D1, D2, and D3 [17]; and primers ITS1/ITS4 and EF1-728F/EF1-986R were used to amplify the sequences of strains A1 through A11 [15]. The PCR amplification reaction mixture consisted of 25 μL of PCR Mix, 2 μL each of forward and reverse primers at 10 μmol/L, 1 μL of fungal DNA, and 20 μL of ddH_2_O to make a total volume of 50 μL. The amplification conditions were set as follows: initial denaturation at 95 °C for 5 min, followed by 34 cycles of 95 °C denaturation for 30 s, annealing at suitable temperatures for 30 s (53 °C for ITS, 52 °C for *tef1*, and 60 °C for *β-tub*), and extension at 72 °C for 30 s. Detect the PCR amplification products using 1% agarose gel. Perform electrophoresis at 120 V for 15 min, then send the DNA samples with correctly sized bands to Sangon Biotech (Shanghai) Co., Ltd. for bidirectional sequencing using the Sanger Sequencing method. A multi-gene phylogenetic tree was constructed using the amplified nucleotide sequences, with *Rhizopus stolonifer* designated as the outgroup and the number of bootstrap replicates was set as 1000. Using MEGA 11 software, a multi-gene phylogenetic tree was constructed employing the Neighbor-Joining (NJ) method.

### 2.6. Determination of the Antifungal Effect of ClO_2_

The susceptibility of pathogenic fungi to ClO_2_ was determined by the mycelial growth rate method. A ClO_2_ slow-release agent (Tianjin Baiduochun Technology Co., Ltd., Tianjin, China) was used to generate a constant supply of ClO_2_ in this study. The concentration of ClO_2_ was detected using the spectrophotometry measurement proposed by Masschelein [26] and then used immediately for experiments. Fungal plugs (Ø = 5 mm) were inoculated in the middle of the medium. The antifungal activity of ClO_2_ was determined using the double-dish counter method [27]. The culture medium was placed in a 28 °C dark incubator for cultivation. After continuous fumigation for 24 h, the diameter of the colonies was measured using the cross method. Each treatment was repeated three times, with three plates per replicate. The colony diameter was measured, and the inhibition rate of different concentrations of ClO_2_ was calculated as follows:

$$Inhibition\;rate(\%)=\frac{\left(Control\;colony\;diameter-Treatment\;colony\;diameter\right)}{\left(Control\;colony\;diameter-Plug\;diameter\right)}\times 100$$

### 2.7. Determination of Fungicide Efficiency

The virulence of fungicides to pathogenic fungi was determined by the mycelial growth rate method according to Chen et al. [28]. Stock solutions of carbendazim, triadimefon, thiophanate-methyl, and prochloraz (Shanghai Macklin Biochemical Co., Ltd., Shanghai, China) were prepared with dimethyl sulfoxide (DMSO), and the stock solutions were diluted to prepare 100 mg/L medicated plates. Fungal plugs (Ø = 5 mm) were inoculated in the middle of the medium. The culture medium was placed in a 28 °C dark incubator for cultivation. The colony diameter was measured, and the inhibition rates of different fungicides were calculated as mentioned above. Each treatment was repeated three times.

### 2.8. Propidium Iodide Staining

After ClO_2_ treatment for 48 h, a small amount of mycelium was taken into a 2 mL centrifuge tube; 200 μL of 10 μg/mL propidium iodide (PI) solution was added for staining. The mixture was stained in the dark at 37 °C for 15 min, washed three times with PBS solution, and the mycelium staining was observed and photographed using a microscope (OLYMPUS BX51, Tokyo, Japan) equipped with a luciferin rhodonine filter set (OLYMPUSU-RFL-T, Tokyo, Japan) at various magnifications. The non-treated group was used as the negative control, and the fungicide was used as the positive control. The experiment was repeated three times.

### 2.9. Effect of ClO_2_ on the Control of Pathogenic Fungi in Fruits

Healthy pear fruits were selected. The surface was then washed with sterile water and subsequently disinfected using 75% alcohol. There were 6 fruits in each group. A small hole (diameter = 5 mm) was made on the equatorial surface of the ‘Akizuki’ pear using a sterile punch. After the pear wound had dried, a 5 mm mycelial plug of each strain was inoculated into the hole with a sterile punch. The samples were placed in a 16 L polypropylene light-shielding freshness maintaining box with a polyethylene bag containing different concentrations of ClO_2_ for the chlorine dioxide fumigation treatment. The concentration of ClO_2_ in the box was measured using spectrophotometry [26]. Fruit without ClO_2_ was used as a control. After continuous fumigation for 24 h, all samples were kept in a constant-humidity incubator (Shanghai PHC Health Medical Devices Co., Ltd., Shanghai, China) at 25 °C. The lesion diameter was measured using the cross method. Each treatment was repeated three times, with three fruits per replicate.

### 2.10. Statistical Analysis

All data were statistically analyzed using the SPSS software version 26.0 (IBM Corp, Armonk, NY, USA). A one-way analysis of variance was undertaken to evaluate the differences between treatments; those followed by different letters are statistically different according to Tukey’s multiple range test for multiple comparison. Significance was declared at *p* < 0.05.

## 3. Results

### 3.1. Isolation and Morphological Identification of Pathogens from ‘Akizuki’ Pear

**Figure 1:**
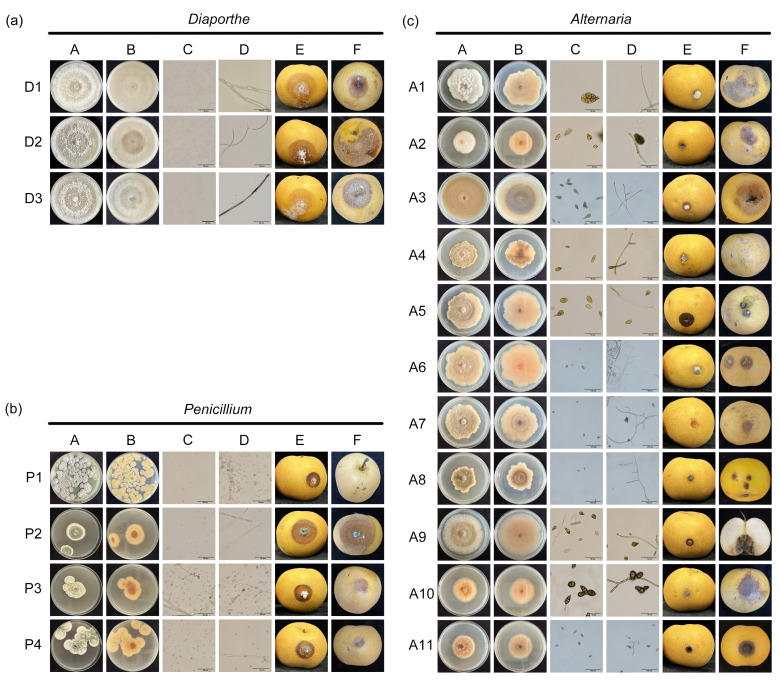
Morphological characteristics of fungal pathogens such as *Diaporthe* (**a**), *Penicillium* (**b**), and *Alternaria* (**c**) in rotten ‘Akizuki’ pears. A, front view of a purified pathogenic fungus colony. B, back view of a purified pathogenic fungus colony. C, conidia morphology, showing a typical characteristic of the pathogens. D, mycelium morphology showing a typical characteristic of the pathogens. E, inoculation symptoms of isolated and purified pathogens. F, original symptoms caused by the pathogen on fruit. The scale bar indicates 50 µm.

By using the tissue separation method, a total of 18 pathogenic fungal isolates were obtained during storage and preliminarily classified into three morphological groups based on colony characteristics (Figure 1). Further, comprehensive microscopic examination of conidial and hyphal structures, coupled with colony morphology analysis, was then conducted for identification of the pathogens at the genus level (Figure 1).

Colonies of isolates D1, D2, and D3 were round and concentric with well-developed mycelia. The color of the colony is initially white and later turns to grayish white, with the production of gray-brown pigments on the surface. Microscopic analysis identified that the conidia of isolates D1, D2, and D3 were single-celled, aseptate, colorless, and transparent, which are morphological characteristics of the genus *Diaporthe* (Figure 1a).

Isolate P1 colonies were radiating, grayish green with raised centers and thick white margins at the edge, and the back of the medium was yellow. The conidia of isolate P1 were subglobose to ellipsoidal, single-celled, aseptate, and greenish. The colonies of isolates P2, P3, and P4 were velvety in texture, blue-green, circular with radial striations, and the reverse side was cinnamon-colored. The conidia of isolates P2, P3, and P4 were oval or round, blue-green, and smooth-walled. Based on these characteristics, isolates P1, P2, P3, and P4 were identified as belonging to the genus *Penicillium* (Figure 1b).

The colonies of isolates A1-A11 were flocculent with dense hyphae, which were white initially and then turned brown, and the back of the medium was cinnamon-colored or brown. The conidia were clavate, ovoid, pyriform, or elliptical with a bluntly rounded base, brown, and two to four septa were observed, which are morphological characteristics of the genus *Alternaria* (Figure 1c).

### 3.2. Pathogenicity Test of Pathogens

**Figure 2:**
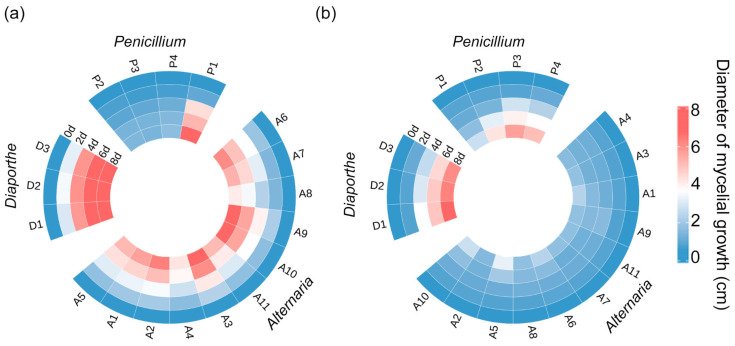
The infecting potential test for representative isolates of different pathogens. Heatmap depicting mycelial growth diameter of each strain on PDA (**a**) and pear fruits (**b**).

Strains of *Diaporthe*, *Penicillium*, and *Alternaria* were inoculated onto healthy pear fruits, inducing disease symptoms in all cases. After 3~5 days of cultivation, obvious disease symptoms appeared, consistent with those observed under natural conditions (Figure 1). Subsequently, the corresponding pathogenic fungi were reisolated from the diseased areas, preliminarily confirming them as the same pathogens. Analysis of lesion sizes from reinoculated fruits revealed that there were differences in pathogenicity among different genera and among different species within the same genus (Figure 2a). The genera *Penicillium*, *Diaporthe*, and *Alternaria*, *Diaporthe* exhibited the strongest pathogenicity. Within *Penicillium*, strain P3 showed strong pathogenicity towards ‘Akizuki’ pears, while strains P1, P2, and P4 exhibited weaker pathogenicity. In the genus *Alternaria*, strain A5 demonstrated strong pathogenicity, whereas strains A7 and A9 showed weaker pathogenicity (Figure 2b). All strains (D1, D2, and D3) in the genus *Diaporthe* exhibited strong pathogenicity. Therefore, strains P3, A5, and D1 were selected based on their pathogenicity for subsequent ClO_2_ concentration screening (Figure 2).

### 3.3. Molecular Biological Identification of Pathogens

**Figure 3:**
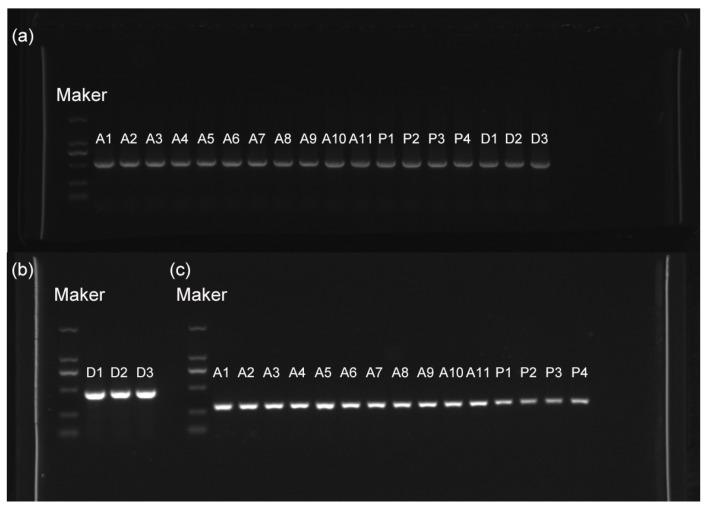
Gel electrophoresis image of PCR-amplified products. (**a**) ITS, (**b**) *β-tub*, and (**c**) *tef1*.

**Figure 4:**
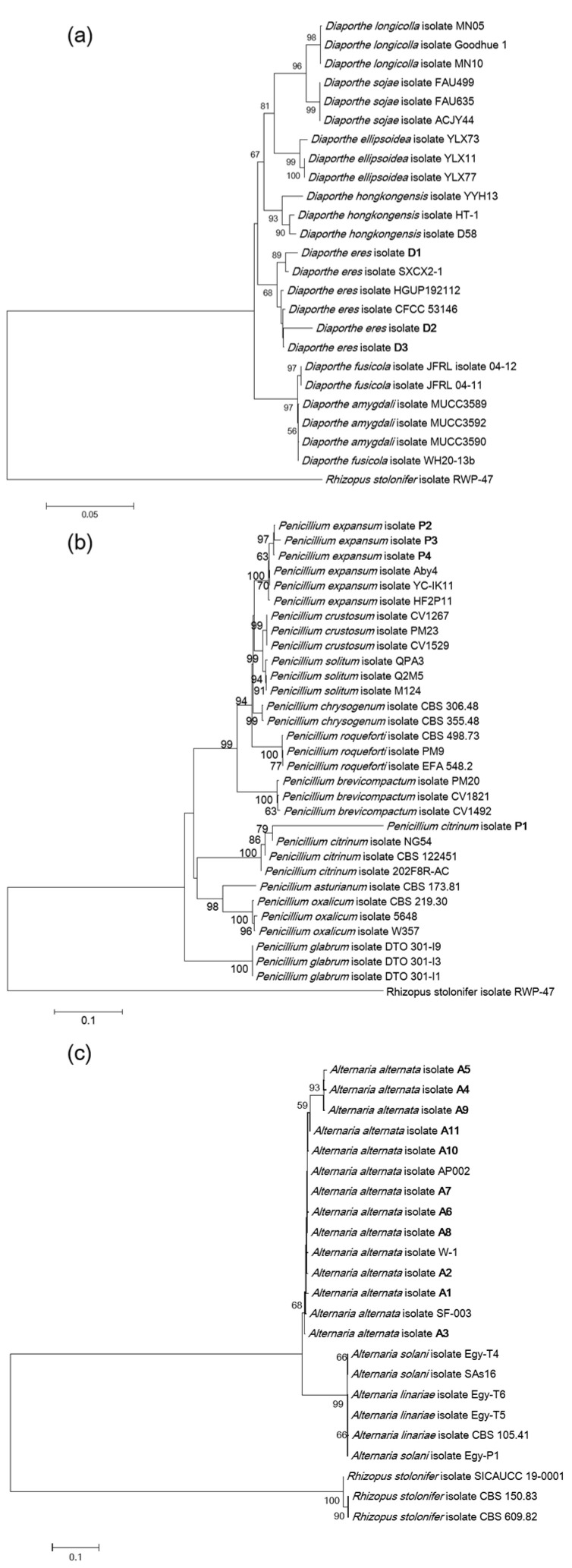
Multi-locus phylogenic tree of the eighteen pathogenic fungal strains based on a Neighbor-Joining analysis. (**a**) Phylogenetic tree based on ITS and *β-tub* gene sequences of strains D1, D2, and D3. (**b**) Phylogenetic tree based on ITS and *tef1* gene sequences of strains P1, P2, P3, and P4. (**c**) Phylogenetic tree based on ITS and *tef1* gene sequences of strains A1-A11. The numbers on the nodes indicate the support percentages of bootstrap. The scales 0.005, 0.1, and 0.1 represent the branch difference length of evolution. The codes following the species names indicate the isolate or strain.

The ITS* (*internal transcribed spacer*)*, *β-tub *(*β-tubulin)*, and *tef1 (*translation elongation factor 1-α*)* regions were amplified from 18 strains by using universal fungal primers, resulting in ITS bands of approximately 500 bp, *tef1* bands around 300 bp, and *β-tub* bands ranging from 500 to 750 bp (Figure 3). The evolutionary tree results showed that strains of the genus *Diaporthe* clustered into ten branches. Strain D1 and *D. eres* SXCX2-1 grouped on the same branch (Table 1), while strain D2 and D3 clustered together with *D. eres* CFCC 53,146 (Figure 4a). Strains P2, P3, P4, and *P. expansum* Aby4 grouped on the same branch with a support rate of 63% (Table 1). Strains P1 and *P. citrinum* NG54 clustered together with a support rate of 79% (Figure 4b). Strains A3, A4, A5, A9, A10, A11, and *A. alternata* AP002 grouped in the same branch, while strains A1, A2, A6, A7, A8, and *A. alternata* W-1 clustered on another branch (Figure 4c). Combining morphological and phylogenetic analyses, strain P1 was identified as *P. citrinum*; strains D1, D2, and D3 as *D. eres*; strains P4, P2, and P3 as *P. expansum*; and strains A1-A11 as *A. alternata*.

### 3.4. Effect of ClO_2_ on the Development of Pathogens

**Figure 5:**
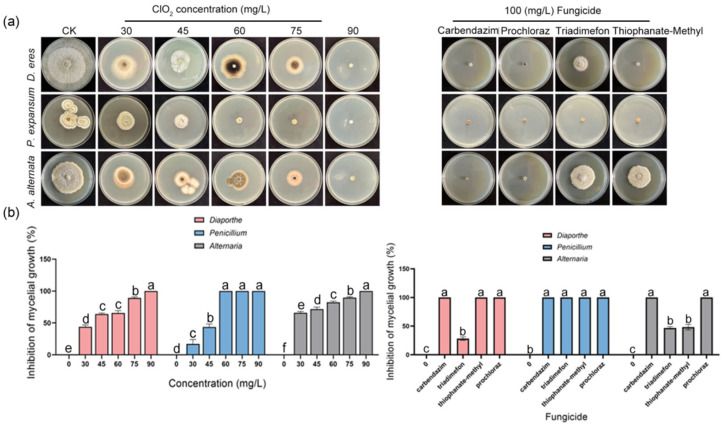
The effect of different concentrations of ClO_2_ and multiple fungicides on mycelial growth of *P. expansum*, *A. alternata*, and *D. eres* in vitro. (**a**) Mycelial growth. (**b**) Inhibition of mycelial growth. Error bars represent vertical bars(*n* = 6 for inhibition of mycelial growth). Different letters indicate significant differences according to Duncan’s test (*p* < 0.05).

To evaluate the antifungal efficacy of ClO_2_, sensitivity assays were performed on *P. expansum*, *A. alternata*, and *D. eres*. As shown in Figure 5a, higher ClO_2_ concentrations significantly reduced lesion diameters and increased inhibition rates. It was demonstrated that ClO_2_ effectively suppresses the development of the postharvest pathogens in ‘Akizuki’ pears. Additionally, pathogens exhibited marked differential sensitivity to ClO_2_ treatment. ClO_2_ had the best inhibitory effect on *A. alternata*, followed by *P. expansum* and *D. eres*. ClO_2_ treatment had an inhibition rate of 17% for *P. expansum* at 30 mg/L and completely inhibited the production of lesions at 60 mg/L (Figure 5b). The dose–effect regression equation was y = 16.0517x − 26.0532, and the EC50 value was 41.98 mg/L (Table 2). ClO_2_ treatment had an inhibition rate of 65% for *A. alternata* at 30 mg/L and completely inhibited it at 90 mg/L (Figure 5b). The virulence regression equation was y = 4.8672x − 6.7795, and the EC50 value was 24.71 mg/L (Table 2). ClO_2_ treatment had an inhibition rate of 44% for *D. eres* at 30 mg/L and completely inhibited it at 90 mg/L (Figure 5b). The virulence regression equation was y = 6.0360x − 9.3617, and the EC50 value was 35.56 mg/L (Table 2).

Furthermore, the antifungal efficacy of ClO_2_ was compared with four commercial fungicides, namely carbendazim, triadimefon, thiophanate-methyl, and prochloraz. At 100 mg/L, all four of the above fungicides significantly inhibited the mycelial growth of *P. expansum*, *A. alternata*, and *D. eres*, whereas ClO_2_ attained complete suppression (100%) at 90 mg/L. This further indicated that ClO_2_ fumigation serves as an effective measure for controlling postharvest diseases in the ‘Akizuki’ pear.

### 3.5. Effect of ClO_2_ on the Cell Membrane of Pathogens

**Figure 6:**
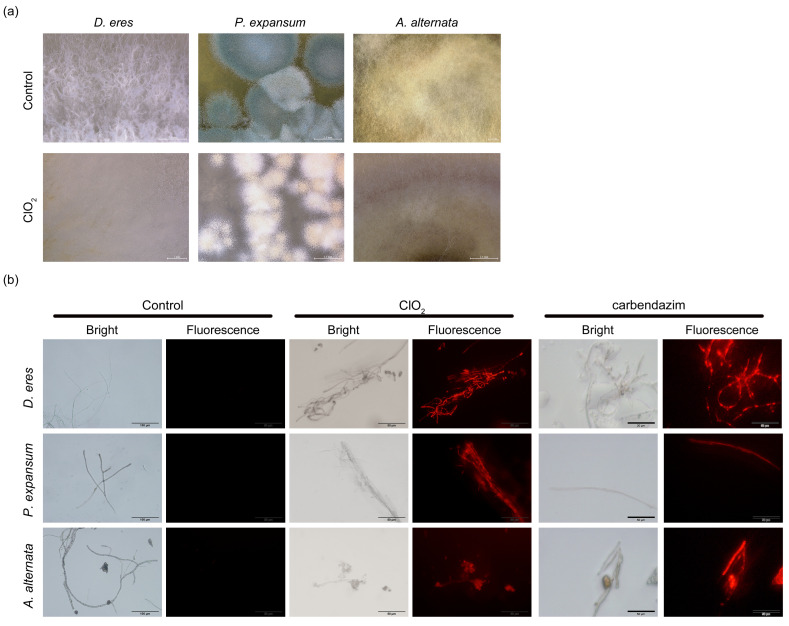
The effect of ClO_2_ on the growth of *P. expansum*, *A. alternata*, and *D. eres*. (**a**) Super-depth-of-field microscope (SDM) images of *P. expansum*, *A. alternata*, and *D. eres* without ClO_2_ treatment and treated with 45 mg/L of ClO_2_. (**b**) Effect of chlorine dioxide treatment on mycelial cell membranes of *P. expansum*, *A. alternata*, and *D. eres*. The scale bar indicates 50 µm.

The impact of ClO_2_ on hyphal morphology and membrane integrity was investigated in *P. expansum*, *A. alternata*, and *D. eres* to elucidate its antifungal mechanism. Based on in vitro experimental results, it was observed that the antifungal activity against pathogenic fungi significantly increased when the concentration of ClO_2_ reached a certain level. A concentration of 45 mg/L ClO_2_ was selected for subsequent experiments to prevent phytotoxicity in ‘Akizuki’ pears while maintaining significant antifungal efficacy. Observation of the hyphal morphology of *P. expansum*, *A. alternata*, and *D. eres* by using ultra-depth-of-field microscopy showed that the color of *P. expansum* hyphae changed from green to white, and hyphal growth was inhibited. The color of *A. alternata* and *D. eres* hyphae changed significantly, with the inhibition of *D. eres* hyphae being the most obvious (Figure 6a). This suggested that ClO_2_ significantly disrupted hyphal morphology in *P. expansum*, *A. alternata*, and *D. eres*, causing abnormal growth and reducing infectivity. Propidium iodide (PI) is a nucleic acid dye that can pass through damaged cell membranes and bind to DNA to form a stable red fluorescent complex. PI staining of hyphae exposed to 45 mg/L ClO_2_ demonstrated red fluorescence in all pathogens, indicating nucleic acid leakage through compromised membranes. *D. eres* displayed the strongest fluorescence intensity (Figure 6b), confirming severe membrane damage. These results revealed that the antifungal mechanism of ClO_2_ involves the disruption of hyphal structure and membrane integrity.

### 3.6. Effect of ClO_2_ on the Disease Incidence of ‘Akizuki’ Pear Fruits

**Figure 7:**
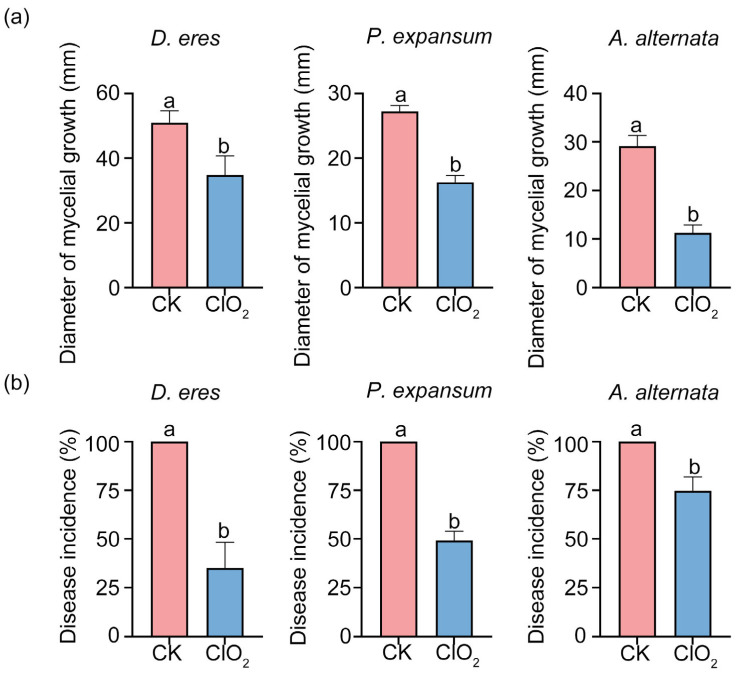
Effect of ClO_2_ (45 mg/L) on lesion length (**a**) and disease incidence (**b**) caused by *P. expansum*, *A. alternata*, and *D. eres* in pears after 3 days of storage at 25 °C. Vertical bars represent standard deviations of the means. Error bars represent vertical bars (*n* = 9). Different letters indicate significant differences according to Tukey’s test (*p* < 0.05).

The pathogenicity experiment confirmed the efficacy of ClO_2_ in suppressing postharvest rot in ‘Akizuki’ pears in vivo. Fumigation with 45 mg/L ClO_2_ significantly inhibited disease progression in pear fruits inoculated with *A. alternata*, *P. expansum*, and *D. eres*. Compared to untreated fruits, the results showed that the lesion diameters of ClO_2_-treated fruits were significantly reduced. The lesion diameter of *P. expansum* was inhibited by 10 mm, *A. alternata* by 18 mm, and *D. eres* by 17 mm (Figure 7a). The disease incidence of ClO_2_-treated pear fruits was reduced by 54%, 73%, and 34% compared with the control group (Figure 7b). The results showed that ClO_2_ fumigation effectively inhibited the occurrence of postharvest diseases of ‘Akizuki’ pear.

## 4. Discussion

Currently, research on the ‘Akizuki’ pear primarily focuses on non-infectious diseases, particularly cork spot, a physiological disorder. For instance, Duan et al. investigated the physiology of cork spot in the ‘Akizuki’ pear by comparing mineral elements and physiological indicators between diseased and healthy fruits [29], and Yang et al. analyzed the mechanisms of cork spot in ‘Akizuki’ pears through combined mineral and metabolomic approaches [30]. In contrast, studies on infectious diseases in the ‘Akizuki’ pear remain limited. This study employed a combination of morphological analysis, multi-gene phylogenetic analysis, and pathogenicity assessment to identify the pathogens, thereby elucidating the primary pathogens involved in the rotting of ‘Akizuki’ pears during storage, specifically within the genera *Alternaria*, *Penicillium*, and *Diaporthe*. Subsequently, effective ClO_2_ concentrations against these pathogens were systematically screened.

Our study found that *D. eres*, *A. alternata*, and *P. expansum* are the most important pathogenic fungi causing postharvest rot diseases in ‘Akizuki’ pears. *D. eres* is known to cause severe fruit rot in pears [31] and diverse phytopathologies including black rot in persimmons [32], root rot in *Coptis chinensis* [33], and postharvest rot in peaches [34]. It is well known that *A. alternata* is a significant postharvest pathogen. The black spot disease, caused by *A. alternata* in pears, not only significantly shortens fruit shelf life but also poses serious food safety risks due to mycotoxin contamination. Furthermore, *A. alternata* demonstrates widely phytopathogenicity, as evidenced by its causation of black rot in cherries [35], leaf spot in passion fruits [36], and scab in kiwifruits [37]. *P. expansum* causes characteristic blue mold decay of fruits across multiple hosts, including kiwifruits [38], apples [39], pears [40], and citruses [41], making it one of the key postharvest diseases. All the pears in our study were collected from Hebei Province, which is one of the major production areas for ‘Akizuki’ pears. However, the identification of postharvest diseases in other major ‘Akizuki’ pear-producing regions in China still requires further research.

In this study, gaseous ClO_2_ fumigation demonstrated concentration-dependent antifungal effects against postharvest pathogens *P. expansum*, *A. alternata*, and *D. eres* in both culture media (in vitro) and fruits (in vivo) (Figure 5a). Complete inhibition was achieved at 60 mg/L for *P. expansum* and 90 mg/L for *A. alternata* and *D. eres* (Figure 5b). These concentrations align with Lee et al.’s findings where 20 mg/L gaseous ClO_2_ completely suppressed *Diaporthe batatas* [42], demonstrating the consistent efficacy of gas-phase treatment. Crucially, efficacy varied substantially across application methods. Semi-permeable film packaging with ClO_2_ slurry achieved complete *A. alternata* control at 10 mg/L in tomatoes [43]. Solid formulations required drastically higher doses than gaseous fumigation. Specifically, effective concentrations reached 400 mg/L for *P. expansum* [23] and 250 mg/L for *P. digitatum* [44]. This dosage reduction underscores the superior efficiency of gaseous fumigation, being attributable to enhanced gas diffusion and sustained biocidal contact. Notably, comparing the same concentration of ClO_2_ with four commonly used fungicides, such as carbendazim, thiophanate-methyl, prochloraz, and triadimefon, the antifungal effect in vitro was similar. The results are consistent with the study by Hatamzadeh et al. [45], who found comparable antifungal effects in vivo and in vitro when comparing ClO_2_ to four commercial fungicides (thifluzamide, imazalil, cyprodinil, and thiophanate-methyl) at 1000 mg/L. Zhang et al. found that treatment with 60 mg/L ClO_2_ can significantly enhance the antioxidant capacity of cherry fruits while effectively delaying the process of quality deterioration [46]. Since the 45 mg/L concentration adopted herein is significantly lower than the above-mentioned safe and effective 60 mg/L, ClO_2_ fumigation will not cause harm to the fruits. Most significantly, gaseous ClO_2_ treatment reduced the disease severity in pear fruits, demonstrating its practical value as an efficient antimicrobial agent (Figure 7a).

The antifungal mechanism of ClO_2_ is currently mainly divided into two categories—by increasing the activity of defense enzymes, and by directly destroying the cell membrane of pathogens to inhibit the development of the postharvest diseases. It is important to note that gaseous ClO_2_ is unstable and sensitive to light and heat, which limits its application [47]. In order to promote the development of ClO_2_ in the fruit preservation industry, studies have explored the effect of different packaging containing chlorine dioxide on the storage quality of grapes and found that ClO_2_ could effectively reduce the respiration rate and significantly increase the total phenols, flavonoids, and anthocyanins compared with the control group [48]. Sun et al. found that ClO_2_ microcapsule treatment could effectively inhibit *Listeria* and *E. coli* and delay the decline in quality during the storage period of blueberries [49]. However, few studies have explored the combination of ClO_2_ with other preservation methods through different packaging approaches to control postharvest diseases. Therefore, it is necessary to further study the combined application of ClO_2_ and other preservation technologies through different packaging strategies.

## 5. Conclusions

This study presents the first systematic identification of pathogenic fungi causing postharvest decay in ‘Akizuki’ pears. The 18 pathogenic fungal strains were identified as *P. expansum*, *P. citrinum*, *D. eres*, and *A. alternata* based on morphological characterization, pathogenicity testing, and multi-locus phylogenetic analysis. Subsequent evaluation of ClO_2_ efficacy demonstrated its potent antifungal activity against the key pathogens *P. expansum*, *A. alternata*, and *D. eres*. The antifungal mechanism involved the disruption of hyphal morphology and damage to cell membrane integrity. Importantly, ClO_2_ exhibits improved antifungal efficacy comparable to that of conventional fungicides. These findings provide crucial insights into the etiology of postharvest decay in ‘Akizuki’ pears and establish gaseous ClO_2_ fumigation as a promising, effective, and eco-friendly strategy for controlling these diseases.

## Figures and Tables

**Table 1:** List of isolates of species used in this study.

Isolates	The Closest Matching GenBank Taxa	GenBank Accession Nos.		
		ITS	*tef1*	*β-tub*
A1	*Alternaria alternata*	PX148910	PX410011	
A2	*Alternaria alternata*	PX148902	PX410009	
A3	*Alternaria alternata*	PX148911	PX410010	
A4	*Alternaria alternata*	PX148904	PX410019	
A5	*Alternaria alternata*	PX148909	PX410018	
A6	*Alternaria alternata*	PX148906	PX410013	
A7	*Alternaria alternata*	PX148907	PX410014	
A8	*Alternaria alternata*	PX148903	PX410012	
A9	*Alternaria alternata*	PX148912	PX410017	
A10	*Alternaria alternata*	PX148905	PX410015	
A11	*Alternaria alternata*	PX148908	PX410016	
AP002	*Alternaria alternata*	OK103568.1	OK120224.1	
W-1	*Alternaria alternata*	MG189596.1	MZ750391.1	
SF-003	*Alternaria alternata*	ON053451.1	ON055374.1	
Egy-T4	*Alternaria solani*	MT996273.1	MT478030.1	
SAs16	*Alternaria solani*	MG525490.1	MG525516.1	
Egy-T6	*Alternaria linariae*	MT996275.1	MT996281.1	
Egy-T5	*Alternaria linariae*	MT996274.1	MT996280.1	
CBS 105.41	*Alternaria linariae*	NR_136092.1	KJ718528.1	
Egy-P1	*Alternaria solani*	MT476733.1	MT478033.1	
SICAUCC 19-0001	*Rhizopus stolonifera*	MN267051.1	MN159909.1	
CBS 150.83	*Rhizopus stolonifera*	AB113022.1	AB512254.1	
CBS 609.82	*Rhizopus stolonifera*	AB113023.1	AB512268.1	
D1	*Diaporthe eres*	PX148848		PX289532
D2	*Diaporthe eres*	PX148846		PX393511
D3	*Diaporthe eres*	PX148848		PX393512
MN05	*Diaporthe longicolla*	OL843914.1		OL999083.1
Goodhue 1	*Diaporthe longicolla*	OL843916.1		OL999081.1
MN10	*Diaporthe longicolla*	OL843919.1		OL999085.1
FAU499	*Diaporthe sojae*	KJ590717.1		KJ610873.1
FAU635	*Diaporthe sojae*	KJ590719.1		KJ610875.1
ACJY44	*Diaporthe sojae*	MW578676.1		MW598122.1
YLX73	*Diaporthe ellipsoidea*	OM538397.1		OM654882.1
YLX11	*Diaporthe ellipsoidea*	OM538389.1		OM654877.1
YLX77	*Diaporthe ellipsoidea*	OM538398.1		OM654879.1
YYH13	*Diaporthe hongkongensis*	PQ049734.1		PP975435.1
HT-1	*Diaporthe hongkongensis*	MT740484.1		MT749776.1
D58	*Diaporthe hongkongensis*	PP383967.1		PP412806.1
SXCX2-1	*Diaporthe eres*	MT877020.1		MT874938.1
HGUP192112	*Diaporthe eres*	MZ724720.1		MZ724004.1
CFCC 53146	*Diaporthe eres*	MN266201.1		MN315471.1
JFRL 04-12	*Diaporthe fusicola*	ON994259.1		OP076826.1
JFRL 04-11	*Diaporthe fusicola*	ON994258.1		OP076825.1
MUCC3589	*Diaporthe amygdali*	OR897081.1		OR913141.1
MUCC3592	*Diaporthe amygdali*	OR913144.1		OR897084.1
MUCC3590	*Diaporthe amygdali*	OR897082.1		OR913142.1
RWP-47	*Rhizopus stolonifera*	MH348275.1		MH370152.1
P1	*Penicillium citrinum*	PX376074		PX373324
P2	*Penicillium expansum*	PX254693		PX373325
P3	*Penicillium expansum*	PX254694		PX393513
P4	*Penicillium expansum*	PX254695		PX393514
Aby4	*Penicillium expansum*	OR426630.1		OL802926.1
YC-IK11	*Penicillium expansum*	MK850332.1		MK862430.1
HF2P11	*Penicillium expansum*	OP178985.1		OP562802.1
CV1267	*Penicillium crustosum*	JX091401.1		JX091537.1
PM23	*Penicillium crustosum*	ON116669.1		ON155603.1
CV1529	*Penicillium crustosum*	JX091538.1		JX091402.1
QPA3	*Penicillium solitum*	MK660355.1		MK675786.1
Q2M5	*Penicillium solitum*	MK660346.1		MK675777.1
M124	*Penicillium solitum*	MK660333.1		MK675764.1
CBS 306.48	*Penicillium chrysogenum*	MH856357.1		
CBS 355.48	*Penicillium chrysogenum*	MH856388.1		JF909948.1
CBS 498.73	*Penicillium roqueforti*	MH860759.1		HQ442359.1
PM9	*Penicillium roqueforti*	ON116661.1		ON155595.1
EFA 548.2	*Penicillium roqueforti*	OK323188.1		OK148549.1
PM20	*Penicillium brevicompactum*	ON116667.1		ON155601.1
CV1821	*Penicillium brevicompactum*	JX091534.1		JX091399.1
CV1492	*Penicillium brevicompactum*	JX091533.1		JX091398.1
NG54	*Penicillium citrinum*	OP464912.1		OP502874.1
CBS 122451	*Penicillium citrinum*	GU944572.1		GU944544.1
202F8R-AC	*Penicillium citrinum*	MZ410306.1		MZ369129.1
CBS 173.81	*Penicillium asturianum*	MH861321.1		KF296470.1
CBS 219.30	*Penicillium oxalicum*	MH855125.1		KF296462.1
5648	*Penicillium oxalicum*	KJ527449.1		KJ527414.1
W357	*Penicillium oxalicum*	MH567091.1		MH593522.1
DTO 301-I9	*Penicillium glabrum*	KM189803.1		KM089053.1
DTO 301-I3	*Penicillium glabrum*	KM189798.1		KM089048.1
DTO 301-I1	*Penicillium glabrum*	KM189797.1		KM089047.1
RWP-47	*Rhizopus stolonifera*	MH348275.1		MH370152.1

**Table 2:** Regression equations of ClO_2_ concentration versus inhibition of *P. expansum*, *A. alternata*, and *D. eres*.

Fungal Species	Regression Equation	EC50 (mg/L)	R^2^
*Alternaria alternata*	y = 4.8672x − 6.7795	24.71	0.918
*Diaporthe eres*	y = 6.0360x − 9.3617	35.56	0.852
*Penicillium expansum*	y = 16.0517x − 26.0532	41.98	0.835

Note: x represents the concentration of ClO_2_ (mg/L), and y represents the fungal inhibition rate (%).

## Data Availability

The original contributions presented in this study are included in the article. Further inquiries can be directed to the corresponding authors.
